# Unveiling conflicting strategies in the Brazilian response to COVID-19: A cross-sectional study using the Functional Resonance Analysis Method

**DOI:** 10.1016/j.dialog.2022.100056

**Published:** 2022-10-06

**Authors:** Alessandro Jatobá, Hugo Bellas, Jaqueline Viana, Paula de Castro Nunes, Raquel Leal, Bárbara Bulhões, Rodrigo Arcuri, Paulo Victor Rodrigues de Carvalho

**Affiliations:** a Centro de Estudos Estratégicos Antônio Ivo de Carvalho (CEE), Fundação Oswaldo Cruz, Rio de Janeiro, Brazil; b Instituto de Medicina Social Hésio Cordeiro (IMS), Universidade do Estado do Rio de Janeiro (UERJ), Rio de Janeiro, Brazil; c Programa de Pós-Graduação em Engenharia de Produção (TPP), Universidade Federal Fluminense (UFF), Niterói, Brazil; d Instituto de Engenharia Nuclear (IEN), Rio de Janeiro, Brazil

**Keywords:** Health systems agencies, Surge capacity, Resilience, Unified health system

## Abstract

By the time the present study was completed, Brazil had been the second epicenter of COVID-19. In addition, the actions taken to respond to the pandemic in Brazil were the subject of extensive debate, since some diverged from recommendations from health authorities and scientists. Since then, the resulting political and social turmoil showed conflicting strategies to tackle the pandemic in Brazil, with visible consequences in the numbers of casualties, but also with effects on the resilience of the overall health system.

Thus, this article explores the actions taken in Brazil to cope with the pandemic from a systems analysis perspective. The structure of the domain was analyzed using Work Domain Analysis, and the activated functions were analyzed using the Functional Resonance Analysis Method, identifying the variability resulting from the conflicting strategies carried out and the consequences to the capacity of the Brazilian health system to respond resiliently to the pandemic.

Results of the study show that functions that overlapped the operation of the overall system were introduced, causing the health system to operate under conflicting objectives, in which functions were created to restrict the outcomes of each other during the entire COVID-19 crisis.

## Highlights

• The Brazilian response to Covid-19 was marked by conflicting strategies in different levels;
• The Functional Resonance Analysis Method (FRAM) was employed to understand the Unified Health System's (SUS) response;
• Studying the Brazilian context promotes significant insights on how health systems are affected by the sociopolitical agenda;
• Conflicting control strategies damped the outcomes of regular functions, undermining the measures taken by health authorities;
• Results point out that the SUS' major aspect for resilience was a long-standing culture for vaccination in Brazil .

## 1 Introduction

The COVID-19 pandemic has been a major challenge to the resilience of health systems. Despite all efforts to respond accordingly, many countries strive to tackle the pandemic. In addition, performing resiliently means more than reacting to crises; it also means being able to sustain regular functioning while coping with extraordinary events. Thus, resilient health systems must be able to adapt to eventual shocks and keep their regular services working in the meantime.

The potential of health systems to perform resiliently has gained additional attention from academia and policymakers since the COVID-19 pandemic [[1], [2], [3]]. Many different issues influenced the struggle of many countries to strengthen the resilient performance of their health systems in the face of COVID-19, such as infrastructure and workforce, mismatches with the political agenda, governance arrangements, and sometimes the unusual interference of organizations and leaderships in and out of government structures.

Health systems are complex and distributed sets of subsystems with many tightly and loosely coupled layers. While coping with the COVID-19 pandemic, these sets of subsystems should have had sufficient and flexible response time, nature, and intensity of responses in order to recover from failures and adaptation, accessing the information they need to manage the control strategies adequately. However, such intrinsic resilient properties of loosely coupled systems — local adaptations and accommodation of failures — have limitations, especially when submitted to conflicting objectives. In addition, a healthcare system normally designed to deal with loose couplings (high response time, low impact, local adaptations, etc.) may be unable to sustain regular performance when sudden changes in the environment tighten a loosely coupled situation, as occurred when the COVID-19 waves strained the health systems.

From a resilience perspective, optimizing work performance to avoid brittleness is essential to systems’ safety. Therefore, safety becomes the result of constant performance adaptation to changing demands. Recent studies found that, much like other sectors [4,5], resilience in healthcare is also built on demand, resulting from adjustments towards overcoming constraints in time and resources in everyday clinical work, rather than the product of rules and procedures [6]. Thus, this paper aims at investigating how the Brazilian Unified Health System (SUS) adapted to the context of COVID-19 using an approach based on systems analysis to uncover the disturbances over the essential functions of the SUS during the pandemic.

The Functional Resonance Analysis Method (FRAM) [7] was conceived as an alternative to accident analysis in complex and adaptive systems like healthcare. It has been widely used for both retrospective [[8], [9], [10]] and prospective [11,12] analysis of systems’ behavior under variable circumstances. In this study, the FRAM contributed to describe the multidimensional nature (e.g., sociocultural, political, biomedical) of sharp-end adaptations and their consequences in the Brazilian response to the COVID-19 pandemic.

By the time this study was carried out, Brazil was the second epicenter of COVID-19 in the world, with approximately 680,000 casualties. The actions taken and their effects over the SUS’s capacity to be resilient to the pandemic have been the subject of significant criticism, especially due to the supposed political dispute concerning the control strategies [[13], [14], [15]]. Thus, in mid-2021 the Brazilian Congress started investigations into the Federal Government’s performance during the pandemic, which uncovered a great amount of data on how the Brazilian Government dealt with the pandemic in different periods.

The Brazilian case is symbolic, not only because of the large numbers of casualties but also for the turbulent implementation of control strategies in different levels of government. Thus, exploring the Brazilian case provides empirical evidence on the effects of political agenda over the sociotechnical performance of health systems, and learning from such experience can improve the future strategies for the next crises.

## 2 Methods

### 2.1 Study design

This is a cross-sectional study, and data collection procedures were based on publicly available information. In late April 2021, the Brazilian Senate established a congressional commission to investigate the actions of the Federal Government during the COVID-19 pandemic. The hearings occurred from early May to late October. Many authorities, politicians, and businesspersons were summoned and heard. The final report of the congressional commission was published on October 26, 2021 [16].

The final report includes information from social media, interviews, newspapers, TV, official statements, as well as the contents of testimonies from different players, including scientists, health authorities and workers, and the investigated persons. The commission conducted an extensive review of documents, publications, and other materials from the legal investigations and coded them into the final report, making it the largest source of information concerning the Brazilian response to the pandemic. Thus, this study takes the final report of the commission as its major source of information.

Latour [17] states that when an specific subject of interest gets into collegiate discussion, the corresponding judgement must also be taken collectively, as matters of fact are always partial, polemical, and political versions of subjects of interest. Thus, this study explores the different aspects of variability in the SUS from actions reported exclusively by the investigation commission, which describes a particular state of affairs, as claimed by Latour.

### 2.2 Work domain analysis

This study employed work domain analysis (WDA) [18] to identify the different system components employed in the response to the COVID-19 pandemic. The resulting description of the domain aims at identifying the implicit purpose and the overall functional aspects that influence the accomplishment of such purpose. The abstraction hierarchy was used as the analysis tool.

The structure of the abstraction hierarchy represents means–end relationships between the elements disclosed in five levels of the system. Moving up the hierarchy focuses on the purposes; moving down focuses on how to carry out those purposes. Higher levels are less detailed than lower levels. Rasmussen recommends modeling activity focused on the span between physical reality and human purposes. Thus, the levels of the abstraction hierarchy represent the explored domain as follows:

● Physical form represents the spatial distribution of matter in the environment, like a portrait of the physical landscape;
● Physical function represents the physical structure of the system and its functional properties;
● Generalized function represents relations among variables across boundaries of physical parts or generic elements of system purposes;
● Abstract function represents the overall function of a system in a generalized causal network, moving in abstraction level independently of the local physical or functional properties;
● Functional purpose represents the observable constraints within the relationship among the variables of the system.

### 2.3 FRAM modeling

The FRAM [7] was chosen as the major coding tool in this study. We used the method to model and analyze the variability in the relations between essential functions of the SUS in the scenarios explored in this study. FRAM models have been extensively used in representing the effects of variability in the functions of complex sociotechnical systems like healthcare [[19], [20], [21], [22]].

The FRAM organizes complex systems in their performed functions rather than their structures, specifying essential functions following the principles of joint cognitive systems [23]. Specifications of FRAM functions are organized into six aspects: inputs, outputs, resources, preconditions, time, and controls. Moreover, functions couple to each other, and such dependencies and interactions are non-linear and influence the propagation of performance variability throughout the system.

## 3 Results

**Fig. 1:**
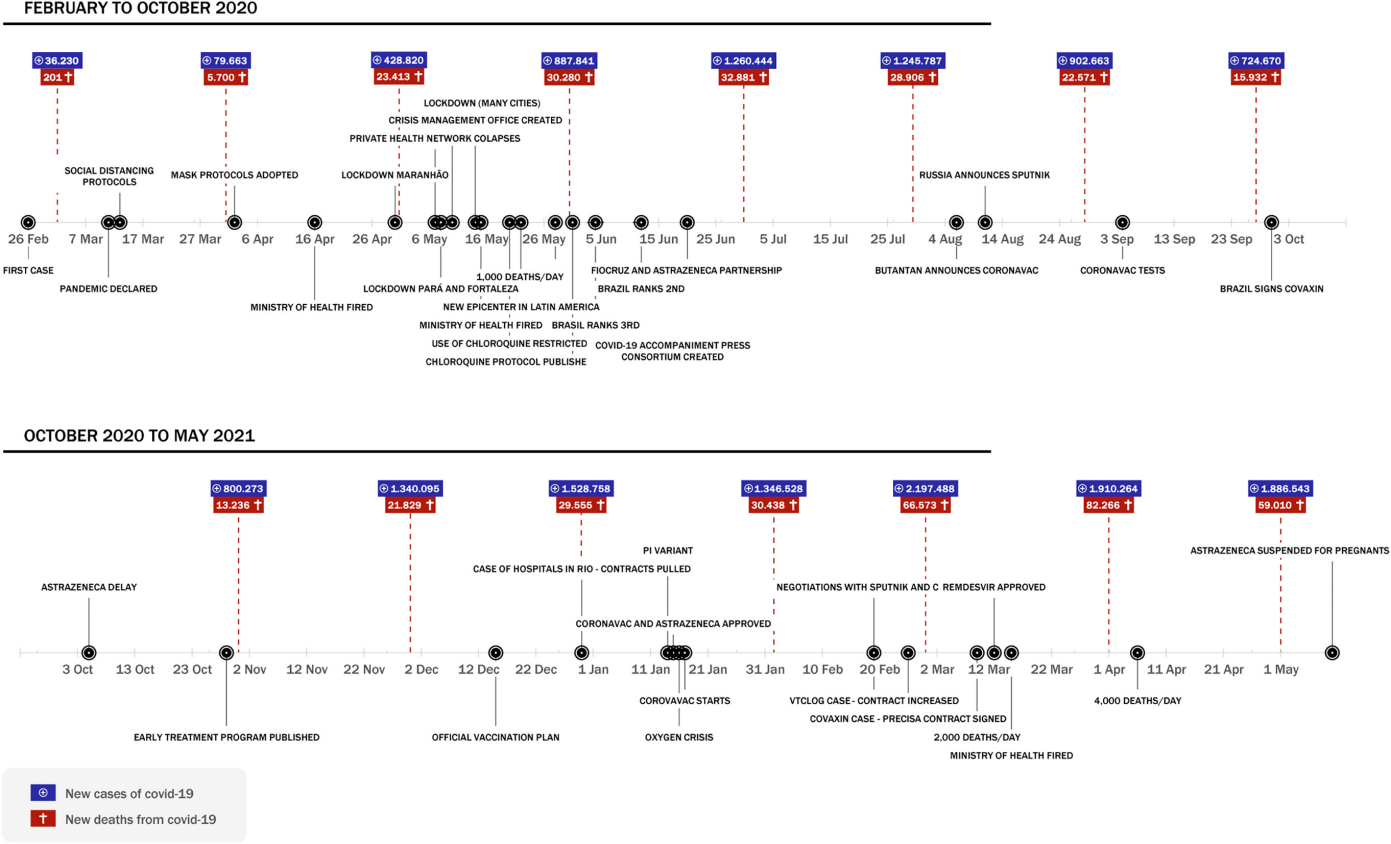
Timeline of the main events during the evolution of COVID-19.

Fig. 1 presents a timeline with the most significant events that occurred in Brazil from the beginning of the pandemic until May 2021, when the investigations began. For data analysis, first the structure of the domain was coded using WDA, and afterwards the activated functions were analyzed using the FRAM.

### 3.1 WDA: how the SUS was structured to tackle emergent events during the COVID-19 pandemic

**Fig. 2:**
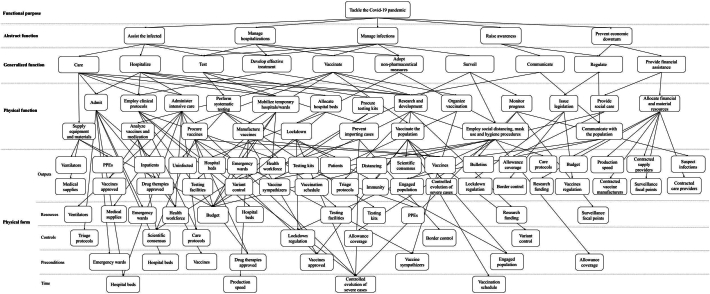
Abstraction hierarchy of the SUS's subsystem to tackle the COVID-19 pandemic.

Considering the infectiousness of the Sars-Cov-2 virus, tackling COVID-19 involves public health measures such as vaccination, non-clinical measures like social distancing and masks, and clinical measures like hospitalization of severe cases. Social distancing affects economic activity, as people must prevent agglomeration, movement, and staying in crowded or indoor spaces. Because non-pharmacological measures affect economic activities, they became an issue on the political agenda. Thus, as Fig. 2 shows, the abstract level of the SUS subsystem dedicated to tackling COVID-19 presents five high-level functions: assist the infected, manage hospitalizations, manage infections, raise awareness, and prevent economic downturn.

The five abstract functions generalized into 10 functions to describe **what** is needed to carry out the abstract purpose: care, hospitalize, test, develop effective treatment, vaccinate, adopt non-pharmaceutical measures, surveil, communicate, regulate, and provide financial assistance. As Fig. 2 shows, the functions relate to the abstract functions in different ways. For example, developing effective treatment is essential for both managing the infections and assisting the infected.

Finally, 21 physical functions worked to describe **how** the generalized functions are accomplished. The physical forms of the listed functions subdivide into outputs, resources, preconditions, controls, and time, to connect the aspects of the domain to the FRAM modeling taken subsequently. Inputs are not graphically represented in Fig. 2 to improve readability and clarity [24].

### 3.2 The FRAM and the variability within the SUS's response to COVID-19

**Fig. 3:**
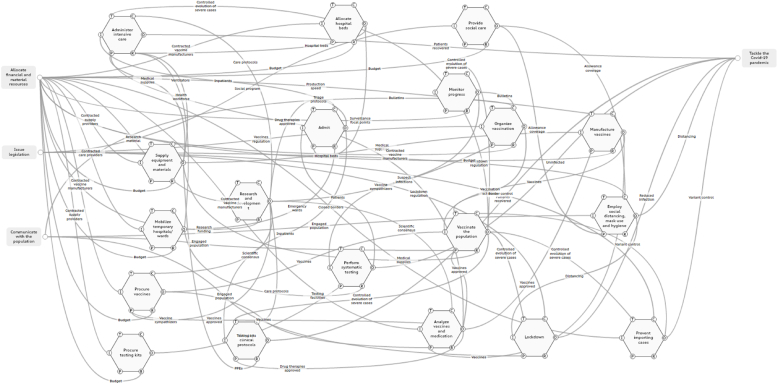
FRAM general model of the health system performance during COVID-19.

The FRAM model in Fig. 3 shows three high-level system functions generating aspects to feed downstream functions: allocate financial and material resources, issue legislation, and communicate with the population. The inputs of these functions come from upper-level functions: communicate, regulate, and provide financial assistance. All these functions relate to the government or other authorities, due to the expected broadness or the financial resources necessary to carry them out accordingly and worked as upstream functions for most functions of the system, as their many couplings demonstrate. Given their importance to the overall functioning of the SUS during the pandemic, these three high-level functions were the basis for the different instantiations, as they were the first major targets of the government's actions.

The models of the three instances presented in this article show symbols in three different colors for clarification. The variability that produced negative results is illustrated in red. The variability that lacked significant effects on the general purpose of the subsystem is illustrated in blue. In addition, resilience relies on the analysis of what goes right, rather than checking only what went wrong when unexpected events occur. The instances in green show the variability that, although unexpected, affected the system positively.

#### 3.2.1 Instantiation 1: allocate financial and material resources

According to the final report of the Congressional Investigation Commission, a “parallel cabinet” of physicians, scientists, and politicians advised the Brazilian Government against social distancing, vaccination, and all types of measures that would supposedly paralyze the economy. That parallel cabinet introduced controls in the function of allocating resources to the system, disturbing its couplings to other important functions, like postponing vaccine acquisition and disregarding manufacturers, narrowing the options of available vaccines.

**Fig. 4:**
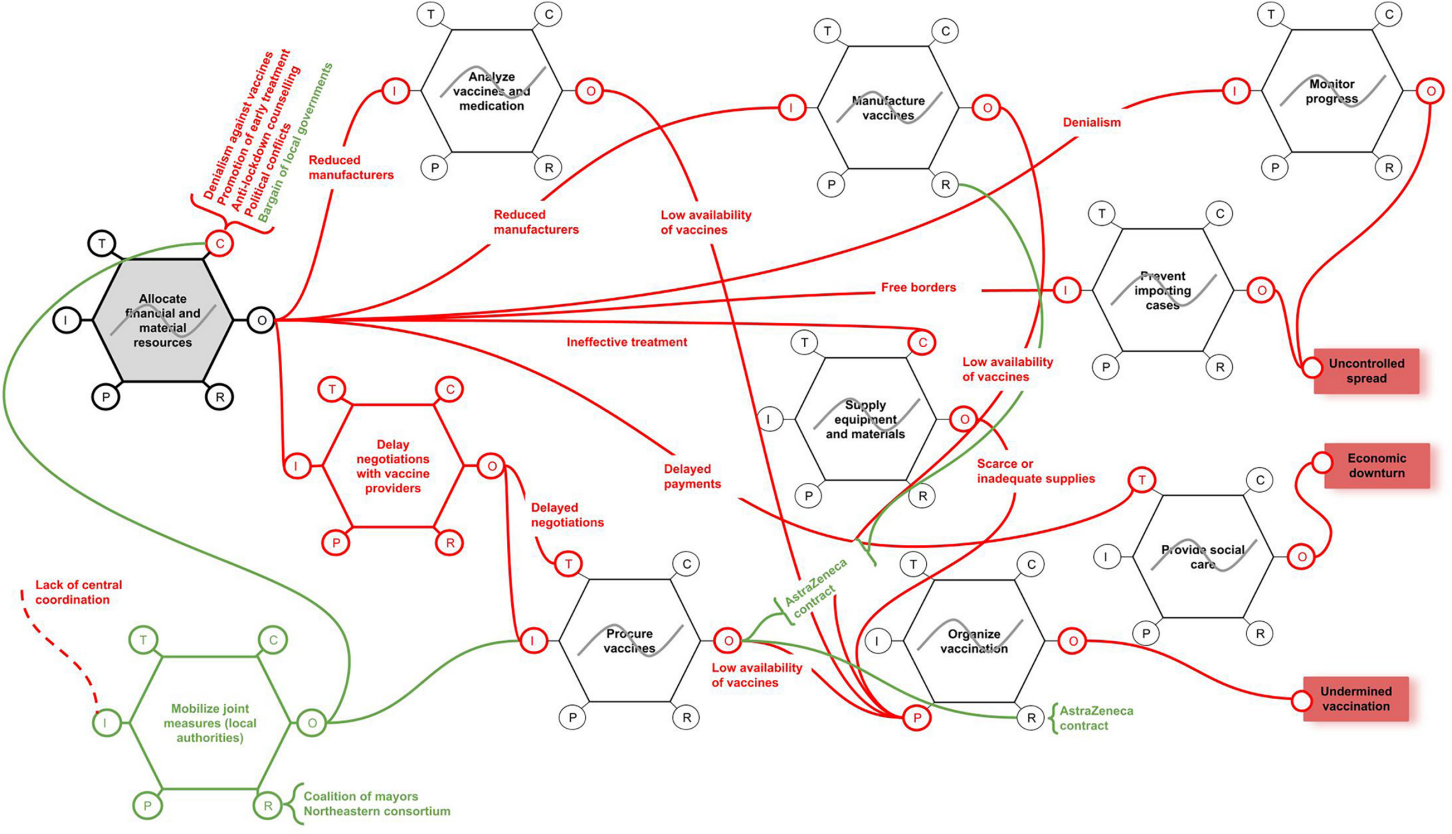
FRAM model of variability in allocating resources to tackle the COVID-19 pandemic. Red: negative variability; Green: positive variability.

This FRAM instantiation on “*allocate financial and material resources*” (Fig. 4) shows the actions taken to delay negotiations with vaccine providers, as described in the CPI report. Fig. 4 presents (in red) a function activated specifically to delay negotiations with vaccine providers. One of the actions performed within this function is described in the report as disregarding the contacts and proposals from the *Pfizer* Company. Actions like these undermined the functioning of the “procure vaccines” function significantly — and its downstream functions — disturbing its outputs and delaying the acquisition of vaccines, reducing the potential of the system to reduce the spread of the disease, and, consequently, hampering its resilient abilities to anticipate and respond. The organization of the vaccination also experienced variability, as the availability of vaccines was affected by the disturbances in the procurement of vaccines. The same effects also resonate in the functions intended to analyze new vaccines and to feed the system with essential supplies.

According to the commission’s report, there was no central coordination to allocate financial and material resources to cope with COVID-19. Thus, many preconditions to organize the vaccination were disturbed by the functional resonance between the “organize vaccination” function and its downstream functions, especially due to the low availability of vaccines. The schedule of vaccination was confused, and the supplies like syringes were eventually missing or wrongly acquired.

An additional expression of erratic measures concerning the allocation of resources regards the promotion of care procedures considered ineffective, according to the investigations. The insistence on ineffective treatment sustained the argument against vaccines and undermined the measures considered effective.

#### 3.2.2 Instantiation 2: communicate with the population

**Fig. 5:**
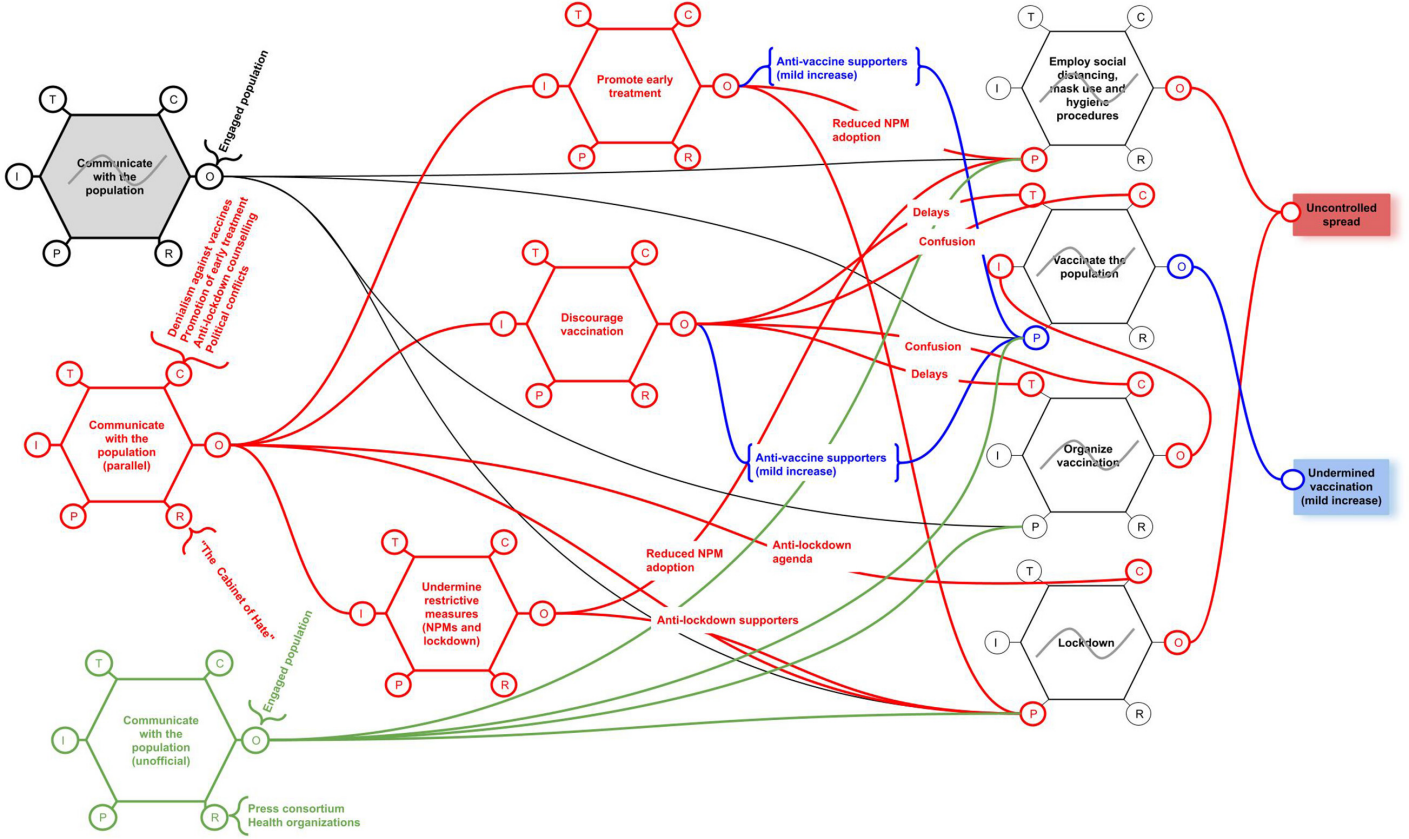
FRAM model of variability in communication with the population to tackle the COVID-19 pandemic. Red: negative variability; Blue: null variability; Green: positive variability.

According to the commission’s report, communication through traditional or social media was employed significantly by the Brazilian Government. In this case, the communication strategy of the Federal Government conflicted constantly with local governments, the press, health authorities, or academia. Fig. 5 shows in red the communications issued by the Federal Government (officially or not) conflicting with the outputs of the communication function coming from the SUS’s officials and other politicians and stakeholders at local (Brazilian states) levels.

Statements in the commission's report evidence the existence of another cabinet for communications, an instance to issue communication to discourage non-pharmacological measures. Thus, the parallel communication strategy was responsible for resonating on the functions intended to prevent the spread of the infection.

Therefore, the Federal Government’s communications strategy was in opposite directions to the evidence-based communications recommended to avoid infections, focused on engaging the population in favor of social distancing, mask use, hygiene habits, and, when vaccines became available, vaccination. According to the information presented in the commission’s report, the Brazilian Government’s communications strategy since the beginning of the pandemic was to advocate for openness of the economy and against social distancing and to promote ineffective therapies. This strategy contributed to decreased awareness of non-pharmacological measures throughout the country [25].

The Federal Government’s agenda advanced, and local governments were pressured by the population, especially as the pandemic progressed and the economy began its downturn. The overall communication strategy included social networks and speeches by high-level authorities. Resignations of two ministers of health occurred in the first six months of the pandemic, supposedly due to disagreements with executive orders.

#### 3.2.3 Instantiation 3: issue legislation

The investigation of the congressional commission points out that the Federal Government took actions to prevent states from ordering lockdowns and social distancing measures during the entire pandemic, even issuing laws and requirements to superior courts. It is noteworthy that there were no actual lockdowns in Brazil. According to the report, the regulation functions were also disturbed by variability caused by the conflicting strategies, which generated popular pressure against restrictive measures and, eventually, against vaccination of certain age groups, like children under 11 years old.

**Fig. 6:**
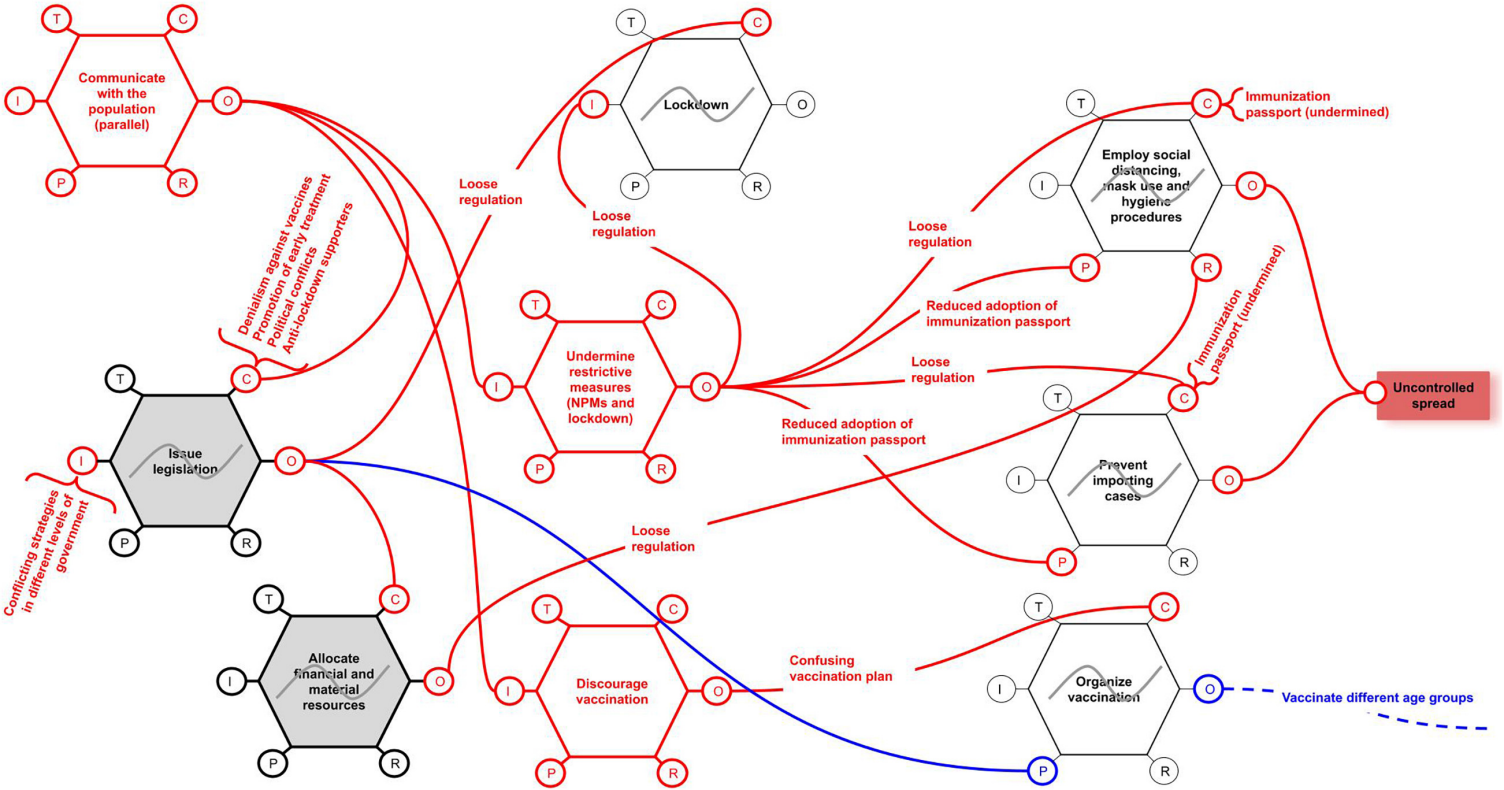
FRAM model of variability in issuing legislation to tackle the COVID-19 pandemic. Red: negative variability; Blue: null variability.

As shown in Fig. 6, the “discourage vaccination” function also resonated on essential aspects to issue legislation, and measures like the immunization passport have been disturbed. For example, the requirement of immunization passports in commercial locations was confusing and hardly supervised by local authorities. Thus, municipal and state-level governments struggled constantly to implement restrictions, and regulations regarding restrictive measures were loose. Legislation regarding the entry of international travelers also experienced difficulties in implementation, as the Federal Government resisted in closing the Brazilian borders.

## 4 Discussion

Prominent literature on complex systems indicates that resilience is a property that systems should develop continuously, on a daily basis [26,27]. This notion of everyday resilience helps explain how systems adapt to adversity while sustaining regular functioning under variable circumstances. However, the COVID-19 pandemic introduced increased political dispute over the implementation of public health functions in Brazil, especially concerning community engagement, funding, acquisitions, and delivery.

Knaul et al. [28] reinforce the importance of nonpharmaceutical measures in tackling the spread of infectious diseases and highlight the influence of local governments in issuing and promoting such measures. The authors indicate that Brazil struggled to coordinate local initiatives with national policies, as the federal, municipal, and state governments rarely agreed on the control strategies to cope with the pandemic. This conflicting scenario undermined the potential of the local health services to perform resiliently, as the results of this study demonstrate.

Analyzing 28 countries, Haldane et al. [3] state that, in general, policies to handle COVID-19 demanded the involvement of different ministries, mobilizing multiple kinds of expertise and evidence-based actions, as previous health disasters like the Ebola surge showed. The most successful countries also articulated temporary advisory groups to support government decisions, despite most of those advisory groups being biomedical rather than multidisciplinary. The results of this study indicate that, in Brazil, such strategies were crossed by a conflicting political agenda that somehow affected their execution.

The FRAM analysis proved to be useful in demonstrating how the actions resonated, generating conflicts. Moreover, it showed the main aspects in such conflicts. The following subsections explore the findings of this study in the context of the broader literature.

### 4.1 The healthcare system operating with conflicting objectives: a crack in Brazil’s federative pact

Once the World Health Organization (WHO) declared the COVID-19 pandemic on March 11, 2020, the Brazilian Federal Government adopted immediate measures to flatten the epidemic curve and to increase the responsiveness of the SUS. Thus, as shown in the FRAM model in Fig. 2, from the second week of March onwards, actions were taken to expand the capacity of the SUS, with an increase in the allocation of hospital beds and human resources, and the issue of specific health protocols. Moreover, regulations regarding telemedicine and telework were also issued, flattening the curve in the first month of the pandemic.

Different studies pointed out the necessity of social distancing to tackle the spread of COVID-19 in Brazil and potential impacts and difficulties in implementing it [[30], [31], [32]]. For most authors, such strategies should be comprehensive and implemented at the national level [3]. In Brazil, the lack of central coordination disturbed different functions in the local health systems, producing multiple unwanted outcomes.

Evidence-based strategies were suddenly abandoned by the end of March 2020, indicating that new (conflicting) objectives were being inserted in the system (see Fig. 3). The government’s communication strategy focused on possible effects on the economy to jeopardize the measures for social distancing advocated by the then minister of health. As Fig. 4 shows, communication strategies were carried out to undermine restrictive measures, relating social distancing to economic downturn and unemployment and stating repeatedly that “the measures to cope with the pandemic would be more harmful than the pandemic itself” [33], hindering the outcomes of nonpharmacological measures, the main function of the health system back then.

Other controversial agendas were carried out, such as the promotion of ineffective treatment, the intensification of the discourse “economy vs. health”, and pressuring state and municipal authorities that advocated for social distancing. Such actions are included in the “undermine restrictive measures” in Fig. 4, and its outputs aimed at reducing the adoption of nonpharmaceutical measures. Expressions of such actions were the “life scoreboard”, in which the number of cured patients was disclosed, obscuring data on the incidence and mortality of the disease; the suspension of press conferences and communication campaigns that had been aired at the beginning of the pandemic; and changes in the publishing process of official data on infections and deaths [34].

Furthermore, strategies to “allocate financial and material resources” struggled as the country faced difficulties in expanding the infrastructure (e.g., the purchase of testing equipment, intubation kits, oxygen, and ventilators), in addition to the Federal Government's attempts to force states and municipalities to loosen social distancing measures, forcing the Brazilian Supreme Court to intervene [35]. This conflict placed more pressure on governors and mayors who faced the closure of non-essential commercial activities while seeking to expand the SUS's responsiveness without the proper financial support.

The Brazilian constitutional model of federalism, characterized by the autonomy of states and municipalities, and the shared responsibility in the articulation of public policies, was undermined by constant confrontation. The Federal Government left aside its prerogatives of issuing regulation, providing technical and financial support, and coordinating collective purchases of vaccines. That fracture in a long-lasting federative pact resulted in confusing directives, disturbing essential functions of the system. To overcome conflicting scenarios, states and municipalities were forced to adopt different kinds of articulations, strengthening horizontal coordination [36].

The mismatch of criteria between the federal and local governments is evident in Fig. 5, especially regarding the legislation for allocating financial resources for the expansion of the SUS's response capacity. Fig. 5 shows the coupling between the “issue legislation” and “allocate resources” functions, as the release of additional funding for states and municipalities disregarded the dynamics of the evolution of COVID-19.

The lack of articulation between federative entities proved to be the main bottleneck. In June 2020, the Ministry of Health decided to stop disclosing information and daily updates on the number of cases and deaths, which prompted different health, social, and press organizations to organize monitoring panels (lethality rate, incidence rate, mortality rate, number of cases, number of deaths, etc.). Fig. 4 shows how different communications were carried out and how their outputs conflicted, disturbing the subsequent foreground functions.

Another expression of constant conflicting objectives concerns vaccination. In December 2020, the government of São Paulo announced the beginning of vaccination with the CoronaVac vaccine, produced by the Butantan Institute in partnership with the Chinese company SinoVac. The Federal Government reacted negatively, as governors from other states even sought partnerships with São Paulo for the purchase of CoronaVac. In fact, the Federal Government had procured the AstraZeneca/Oxford University vaccine, but it was not available at the time CoronaVac was announced (see colored green in Fig. 3).

Moreover, states and municipalities joined forces to face the lack of central coordination, holding meetings to discuss joint measures to fight COVID-19 and bargaining the purchase of vaccines in negotiations with providers. For example, the “Northeastern Consortium”, created in August 2019, became a center for discussion concerning the actions taken in the northeast region of Brazil. In the other states of the federation, most governors and mayors continued to adopt independent measures to combat the pandemic, following recommendations from the WHO and the scientific community.

Despite successive changes of health ministers, conflicts between the Federal Government and local governments have not ceased. In May 2021, after the vaccination of teenagers was declared safe, new conflicts emerged concerning the vaccination calendar. The crisis peaked in mid-September 2021, when the Ministry suspended vaccination for teenagers without comorbidities, allegedly because states and municipalities are not according to the national vaccination plan, or due to mistrust in the safety of vaccines for the referred age group [37].

### 4.2 Effects of poor prediction of pandemic behavior on control strategies

Different social distancing policies have been implemented worldwide, according to the dynamics of SARS-CoV-2 in different countries. In general, those policies were formulated from evidence-based simulation models, some derived from well-known optimal control algorithms [[38], [39], [40]]. Control strategies based only on trial and error are risky in an epidemic context. Thus, Join et al. [41] state that policymakers should focus on more rigorous but realistically constrained approaches when uncertainties blur the known parameters for social distancing measures.

Despite the SUS being entitled to the largest health programs, and the consensus that well-designed social distancing policies were essential to mitigate the spread of the pandemic, to prevent the overload of the health services, and to reduce the economic downturn, the Brazilian Federal Government was reluctant to implement nationwide control strategies. In fact, the report of the investigation commission indicates that the Federal Government discouraged nonpharmaceutical measures, claiming they were erroneous and that they would be responsible for economic downturn.

Initiatives in the application of discrete control algorithms to support optimal control strategies were successful in predicting the complex behavior of SARS-CoV-2, but, in Brazil, such initiatives seemed limited and without central coordination [42,43]. Although the complexity of the factors involving the pandemic prevents complete forecasting of the virus’s behavior, anticipation was essential for the health system’s resilience, and consistent control measures could avoid confusing social distancing, even reducing the necessity of extensive lockdowns.

### 4.3 Rebound to the vaccination culture as a form of resilience

The SUS stands out for its broad and universal coverage. It covers the entire Brazilian population, but as private healthcare providers also operate in Brazil, the services of the SUS are used mostly by the poorest (i.e., most Brazilians). However, services like vaccination are used by the entire population for most of the available vaccines, including the newly released COVID-19 vaccines.

In Brazil, a pro-vaccination culture seems to be well established, as vaccination against COVID-19 advanced quickly, even in the face of conflicts between government levels and delays in the acquisition and distribution of vaccines. Some literature relates the advances in vaccination against COVID-19 to the fact that the Brazilian national immunization program has national and international recognition for promoting free vaccination of more than 15 immunogens, and it continues to expand and update, ensuring universal and integral coverage [44,45].

However, in March 2021, after the Supreme Court authorized the purchase of supplies and vaccines by states and municipalities, and given the inertia and difficulties generated in the purchase and distribution of vaccines and supplies by the Federal Government, a coalition of mayors created a national consortium for the purchase of vaccines and supplies. The measure, together with public opinion and an increasingly unfavorable political environment for the Federal Government, made negotiations with pharmaceutical companies occur more rapidly. Even so, several times the supply of vaccines to states and municipalities was interrupted, which directly affected vaccination coverage.

In this case, it is important to note that the “vaccination culture” of the Brazilian population, developed over decades of successful vaccination campaigns against multiple diseases, damped the outcomes of several functions included in the system to discourage or delay the vaccination process. Brazil has a history of successful vaccination campaigns, such as for polio in the 1970s and 1980s, smallpox in the 1990s and 2000s, and, recently, yellow fever.

## 5 Conclusions and further work

Highlighting resilience and brittleness relies strongly on in-depth understanding of systems' functioning. In the case presented in this article, many of the strategies adopted were implicit, and information sources were scarce. Moreover, the actions taken to cope with COVID-19 in Brazil involved many functions within the healthcare domain, especially regarding the conflicts between government, technicians, and the demands of the population.

Methodologically, the WDA presented in this paper proved to be useful to detail the functional structure of the subsystem of the SUS dedicated to tackle the COVID-19 pandemic in Brazil. The WDA’s different levels enabled the understanding of how the healthcare system is organized from policy to action, in a loosely coupled set of intricate subsystems and functions. In addition, the lower levels of physical function and physical form could be translated into FRAM functions and aspects, clearing the couplings between functions and, consequently, the potential variability within the system, and most importantly, showing how conflicting objectives operate when functions are created to restrict the outcomes of others.

Thus, there are three core findings in this study that demonstrate the potential of systems analysis with the FRAM to unveil instances of resilience and fragility in health systems operating under crisis and, therefore, indicate possibilities for policymaking toward strengthening decentralized systems’ response to future disruptive health events:

• decentralization of policymaking entailed conflicting strategies, as poor articulation undermined the elaboration of evidence-based nationwide protocols;
• adequate response relies on acute monitoring and anticipation, and learning from shared experiences is essential for overall functions;
• the dynamics of the health systems were affected by local ideology, as the outputs of public health functions vary according to the responses of local players.

The evidence presented in this article might help managers and policymakers in the health sector to manage the complex interplay of actions by the agents that operate health systems under crisis, fostering innovative control strategies to handle future disruptive events.

## Author contribution statement

This study was led by the ResiliSUS Lab, Antônio Ivo de Carvalho Center for Strategic Studies, Oswaldo Cruz Foundation, Brazil. Paulo Victor de Carvalho and Alessandro Jatobá worked on the general conceptualization, data collection, and data analysis, and were responsible for the overall writing of the manuscript. Paula Nunes, Hugo Bellas and Jaqueline Vianna presented a theoretical background and discussed the results. Raquel Leal produced the models and graphics presented in the fig.s. Rodrigo Arcuri and Bárbara Bulhões coded the data and reviewed the results.

## Declaration of Competing Interest

The authors have no competing interests to declare.
